# Hidradenitis suppurativa and daily lifestyle

**DOI:** 10.3389/fimmu.2025.1627638

**Published:** 2025-06-27

**Authors:** Natsuko Saito-Sasaki, Yu Sawada

**Affiliations:** Department of Dermatology, University of Occupational and Environmental Health Japan, Kitakyushu, Japan

**Keywords:** hidradenitis suppurativa, lifestyle factors, smoking, diet, physical activity

## Abstract

Hidradenitis suppurativa (HS) is a chronic, relapsing inflammatory skin disorder that significantly impairs quality of life. While its pathogenesis involves genetic, immunologic, and environmental components, emerging evidence highlights the substantial impact of modifiable lifestyle factors on disease onset and progression. This review summarizes the current understanding of how lifestyle-related elements interact with the inflammatory milieu in HS. Beneficial factors, such as short-chain fatty acids (SCFAs), omega-3 fatty acids, and vitamin D, play a protective role by suppressing proinflammatory cytokines, enhancing antimicrobial responses, and promoting the growth of beneficial gut microbiota. Diets rich in vegetables, fruits, chicken, and fish are supportive in maintaining immune balance. Conversely, detrimental factors, including smoking, alcohol consumption, obesity, and sleep disorders, are associated with heightened inflammatory responses, increased disease severity, and higher recurrence rates. Understanding these lifestyle-related influences provides a foundation for non-pharmacologic, adjunctive interventions aimed at mitigating disease burden. Comprehensive management of HS should therefore include lifestyle modification as a key component to improve patient outcomes.

## 1 Introduction

The skin is the outermost organ and serves as the body’s first line of defense (1, 2). It is constantly exposed to a multitude of external factors (3), including ultraviolet radiation (4), bacteria (5), and mechanical stress (6). As an immune organ, the skin plays a critical role in initiating immune responses to these external stimuli, thereby protecting the host (7, 8). This unique position makes it highly susceptible to environmental influences, particularly those involving external and lifestyle-related factors. Consequently, inflammatory skin conditions, such as psoriasis and atopic dermatitis, are significantly influenced by these external stressors and immune-related mechanisms (9).

Hidradenitis suppurativa (HS) is a chronic inflammatory skin disorder characterized by the formation of painful nodules and abscesses, primarily in intertriginous areas, with the disease originating in the hair follicle (10). These include regions subjected to mechanical stress, such as the axillae, groin, buttocks, and perineal areas (11, 12). HS imposes a substantial burden on affected individuals, not only due to its physical symptoms but also because of its significant impact on quality of life (13). Hidradenitis suppurativa is fundamentally an inflammatory disease caused by immune dysregulation, with environmental factors such as mechanical friction contributing to disease progression (14, 15). Bacterial colonization commonly occurs as a secondary event and may exacerbate chronic inflammation and abscess formation.

Recent studies have underscored the role of lifestyle factors in the development and progression of HS. Smoking, for instance, has been strongly linked to disease severity, potentially due to its pro-inflammatory effects and adverse impact on immune regulation (16). Similarly, dietary habits such as diets rich in saturated fats are thought to exacerbate inflammatory pathways (17).

This review aims to bridge the gap between understanding the fundamental mechanisms underlying HS and the role of modifiable lifestyle factors. By highlighting the intricate relationship between these elements, we provide insights that support the development of comprehensive and individualized management strategies for patients with HS. Emphasizing the importance of lifestyle interventions, this review aspires to contribute to improved care and quality of life for those affected by this challenging condition.

## 2 Pathogenesis of HS

HS is currently understood as an inflammatory disease with the hair follicle as the central pathological unit. Hair follicle blockage leads to cysts, abscesses, and the formation of tunnels under the skin (18). In chronic stages, B cells and plasma cells infiltrate the tissue (19). Early inflammation involves neutrophils, macrophages, and dendritic cells. These cells show increased TLR2 expression in response to microbial components (19). Macrophages produce IL-12 and IL-23, leading to Th17 cell infiltration and further inflammation (20).

Inflammatory cytokines such as TNF-α, IL-1β, IFN-γ, IL-12, IL-17A/F, IL-23, and IL-36 are highly expressed in lesions (14, 21). In particular, TNF-α and IL-17A/F are key therapeutic targets in HS, with biologics targeting these cytokines showing high efficacy in reducing inflammation (22, 23). In addition, matrix metalloproteinases (MMPs), secreted by neutrophils and fibroblasts, play a key role in tissue destruction and sinus tract (tunnel) formation (14). IL-36 induces IL-8, contributing to the inflammatory process (24). Antimicrobial peptides like β-defensin-2, psoriasin, and cathelicidin are also elevated (25). These factors cause tissue damage, while TNF or IL-17 inhibitors can reduce inflammation (22, 26).

## 3 The association between lifestyle and inflammatory skin disorders

Inflammatory skin disorders represent a significant burden on affected individuals. Increasing evidence highlights the profound influence of lifestyle factors—such as smoking, diet, physical activity, and sleep—on the development, progression, and management of these conditions. These modifiable factors not only exacerbate systemic inflammation but also interact with underlying immunological pathway, underscoring their critical role in both the pathogenesis and treatment of inflammatory skin diseases. These findings suggest that targeted lifestyle modifications could serve as valuable adjuncts to traditional medical therapies, offering a holistic approach to improving patient outcomes.

This review explores the intricate relationships between lifestyle factors and inflammatory skin disorders, focusing on key modifiable elements such as smoking, diet, physical activity, alcohol consumption, and sleep. By synthesizing current evidence, we aim to provide a comprehensive overview of how lifestyle adjustments can complement medical interventions in the management of these difficult conditions.

### 3.1 Smoking

Several clinical studies have identified the significant impact of smoking on the risk of HS (16). A study conducted on a representative sample of the French population (n=10,000) demonstrated that current smoking is a significant risk factor for hidradenitis suppurativa (HS) (16). Multivariate analyses revealed a strong association between self-reported HS and current smoking, with an odds ratio of 4.16 (95% confidence interval (CI): 2.99–8.69). This finding underscores the critical role of smoking as a modifiable risk factor in the development and progression of HS.

In addition, the severity of HS is also influenced by smoking. Ordinal logistic regression was used in 846 consecutive Dutch patients with hidradenitis suppurativa to calculate Odds ratio (OR) for severity according to Hurley. Severity was associated with smoking pack-years (OR;1.02) (27). In another study of 171 patients with HS, complete epidemiological data were available for 133 individuals (28). The majority individuals were current smokers (77%) or former smokers (7%). Smoking showed a strong correlation with the number of affected body areas. Multiple logistic regression analysis revealed that current or former smokers had a significantly increased risk of having more than two affected body areas compared to non-smokers (OR: 3.56, 95% CI: 1.27–9.96). These findings highlight that smoking, especially active smoking, is closely linked to greater disease burden in HS.

### 3.2 Diet and nutrition

Emerging evidence suggests a connection between certain dietary patterns and inflammatory skin disorders. A cohort of 320 HS patients undergoing TNF inhibitor treatment was established to collect clinical data, including diet and lifestyle, alongside biological samples (17). Surveys among HS patients revealed that 32.6% identified symptom-exacerbating foods, with sweets (67.9%), bread/pasta (51.1%), dairy (50.6%), and high-fat foods (44.2%) most commonly cited. Conversely, alleviating foods included vegetables (78.7%), fruit (56.2%), chicken (51.7%), and fish (42.7%). These findings suggest dietary counseling could benefit HS management.

### 3.3 Dietary fiber

Dietary factors affecting the gut microbiome play a critical role in the pathophysiology and management of HS. Among these, dietary fiber has garnered attention due to its potential to modulate gut dysbiosis and systemic inflammation. The gut microbiota ferments dietary fibers to produce short-chain fatty acids (SCFAs) (29), such as acetate, propionate, and butyrate, which have anti-inflammatory properties and promote metabolic homeostasis. SCFAs are known to stimulate adipose tissue to upregulate fat oxidation, contributing to weight loss and reduced systemic inflammation. In HS, these mechanisms may alleviate disease severity by counteracting the pro-inflammatory milieu driven by obesity and dysbiosis. Notably, skin bacterial dysbiosis is a hallmark of HS, with a significant reduction in beneficial commensals such as *Cutibacterium* and an overgrowth of anaerobic pathogens like *Peptoniphilus* and *Porphyromonas* (30). Restoration of microbial balance through dietary fiber intake may help mitigate this dysbiosis.

Alterations in gut microbiota composition have been documented in HS and other inflammatory conditions. A pilot study investigating the effects of adalimumab, a systemic anti-inflammatory therapy, revealed shifts in gut microbiota composition and function after 12 weeks of treatment (31). Responders to adalimumab therapy exhibited increased levels of SCFAs, particularly acetate and propionate, along with an enrichment of beneficial gut bacteria such as *Prevotella* species and *Faecalibacterium prausnitzii*. However, no direct correlation between SCFA levels and clinical response, as assessed by HiSCR, was observed in the study.

Recent studies have highlighted the potential of dietary interventions to influence HS outcomes. For instance, low-carbohydrate and high-fiber diets have been associated with significant weight loss and clinical improvement in HS patients (32). One case series reported marked remission in patients adhering to a structured low-carbohydrate, high-fiber dietary regimen, with concurrent improvements in microbiome diversity and reductions in inflammatory markers. These findings underscore the importance of dietary fiber as a modifiable factor in HS management.

### 3.4 Fatty acids

Recent research highlights the significant role of fatty acids in HS pathophysiology and potential therapeutic strategies. Lipid mediators derived from omega-3 and omega-6 PUFAs regulate cutaneous homeostasis and inflammation (33, 34). In HS, the lipid mediator profile is disrupted, with increased levels of pro-inflammatory leukotriene B4 (LTB4) and reduced anti-inflammatory metabolites derived from the 15-lipoxygenase (15-LO) pathway which is anti-inflammatory lipid mediators produced by the enzyme 15-lipoxygenase (15-LO), play a pivotal role in resolving inflammation (35). LTB4, produced through the hyperactivation of the 5-lipoxygenase (5-LO) pathway in macrophages, contributes to neutrophil recruitment and activation, exacerbating inflammation in HS lesions. These lipidomic alterations underscore the role of lipid mediators in driving the chronic inflammatory process in HS.

The role of dietary fatty acids in HS severity has also been explored. A case-controlled study examining adherence to the Mediterranean diet (MD) and body composition revealed that HS patients with poor adherence to MD and unfavorable body composition experienced more severe disease. Lower levels of n-3 PUFAs were associated with increased disease severity of HS Sartorius score. These findings suggest that dietary modifications to enhance fatty acid intake, particularly n-3 PUFAs, may ameliorate HS symptoms (36).

### 3.5 Mechanical stress

HS is exacerbated by mechanical stress, which plays a critical role in its pathogenesis (6). Structural and functional abnormalities in the folliculopilosebaceous unit (FPSU), such as follicular hyperkeratosis and infundibular hyperplasia, may predispose HS patients to follicular occlusion and rupture under mechanical stress. Friction not only thickens the epidermis by promoting keratinocyte differentiation and proliferation but also causes retention of hair follicle debris, further aggravating the condition.

Beyond physical effects, mechanical stress activates an inflammatory cascade by increasing matrix metalloproteinase 9 levels (37). In particular, intertriginous regions are the most common in HS. Tight-fitting undergarments, which increase skin pressure and friction, further intensify inflammation. Although loose and breathable clothing is recommended to minimize these effects, addressing the underlying mechanical and immunological triggers is paramount in managing HS effectively. By targeting mechanical stress and the resulting cytokine-driven inflammation, therapeutic strategies can mitigate disease severity and progression.

### 3.6 Obesity

Obesity is a well-recognized risk factor for various inflammatory diseases, including psoriasis. This association is largely attributed to chronic low-grade inflammation, which contributes to immune dysregulation and disease exacerbation. Similarly, HS has been strongly linked to obesity. Several factors contribute to this increased risk. The average BMI of HS patients in studies is consistently higher than that of controls (27.5 ± 6.3 kg/m² vs. 25.9 ± 5.6 kg/m²) (38). Indeed, previous study demonstrated further evidence supporting an increased risk of HS by obesity (39).

Obesity exacerbates amplifying pro-inflammatory cytokines such as IL-17 (40), thereby aggravating skin damage and encouraging flare-ups. The combination of HS and obesity also correlates with increased unemployment rates, further compounding the disease burden.

Despite the well-established association between obesity and HS, referral rates to weight-management services remain alarmingly low. A retrospective study of 50 HS patients revealed that 84% were overweight or obesity, yet only 12% were referred to weight-management programs by primary care providers (41). This lack of referrals underscores the need for increased awareness among healthcare professionals regarding the benefits of weight reduction in reducing HS severity and mitigating associated cardiovascular risks. Weight loss, whether achieved through lifestyle changes or bariatric surgery in severe cases, has been shown to reduce disease activity, decrease flare frequency, and improve overall outcomes for HS patients.

### 3.7 Vitamin D

Several studies have identified that HS patients exhibited vitamin D deficiency (42). One study reported a significant inverse correlation between serum vitamin D levels and disease severity (43). Mean vitamin D levels were consistently lower in HS patients compared to controls, such as 8.4 ng/mL vs. 17.6 ng/mL) (44). Studies on supplementation reported improvements in clinical severity, with one showing a 75% median reduction in nodules after supplementation. A randomized controlled trial is needed to confirm these findings.

Vitamin D plays a role in regulating the immune system and has anti-inflammatory effects, which could be beneficial in HS. It induces the production of antimicrobial peptides, modulating inflammation in skin conditions (45). Additionally, vitamin D is suggested to suppress overexpression of hypoxia-inducible factor-1α (HIF-1α) (46), which is elevated in HS patients and contributes to keratinocyte hyperproliferation and IL-17 production (47). These findings suggest that vitamin D supplementation might help mitigate HS symptoms, although randomized controlled trials are necessary to confirm its therapeutic role.

### 3.8 Physical Activity (PA)

Hidradenitis suppurativa (HS) is influenced by lifestyle factors such as physical activity. A previous study aimed to explore the association between adherence to a Mediterranean diet, physical activity, and HS severity. A cross-sectional study involving 221 HS patients revealed that physical activity plays a significant role in disease management. Both self-reported disease severity and IHS4 scores showed an inverse association with the intensity of physical activity, highlighting its potential to alleviate HS symptoms (48). Combined with regular physical activity, the benefits appeared to be even more pronounced in MD. Promoting a physically active lifestyle alongside an MD could serve as a powerful, non-pharmacological approach to improving outcomes in HS patients.

A Cohort study in Northern Netherlands investigated the relationship between physical activity and HS prevalence, development, and severity. A nested case-control design included 1,004 HS patients and 5,000 age-matched controls. HS patients had significantly lower PA levels compared to controls (49). Reduced physical activity was strongly associated with the prevalence and development of HS, underscoring the potential role of inadequate PA in disease progression. Lower physical activity levels may contribute to systemic inflammation, exacerbating HS severity and its immune-mediated inflammatory pathways. These findings emphasize the critical role of PA in HS management and prevention. Promoting increased physical activity levels could mitigate HS severity and reduce its prevalence, offering a practical intervention strategy for HS.

### 3.9 Alcohol

Increased alcohol consumption is significantly associated with a higher rate of HS recurrence following wide excision surgery (50). This relationship underscores the role of lifestyle factors in the long-term outcomes of HS management.

Another study reveals a significant association between HS and substance use disorder (SUD), with alcohol being the most common substance misused among HS patients (51). Of the HS patients identified with SUD, 47.9% (630/1,315) reported alcohol misuse. Compared to patients without HS, those with the condition had significantly higher odds of SUD (adjusted odds ratio: 1.50, 95% CI: 1.42–1.59), further highlighting the need to address alcohol use in this population.

Alcohol-induced increases in inflammatory cytokines (52), such as TNF-α and IL-6, may exacerbate the chronic inflammatory environment characteristic of HS. These findings highlight the importance of preoperative counseling for patients, with an emphasis on reducing alcohol intake to lower the risk of recurrence.

### 3.10 Sleep disorder

Increasing evidence indicates a strong association between HS and sleep disorders, particularly obstructive sleep apnoea (OSA), with shared risk factors and overlapping inflammatory pathways playing central roles.

OSA is significantly more prevalent in individuals with HS compared to the general population. A population-based study involving 4,417 HS patients and 22,085 matched controls demonstrated that 8.8% of HS patients had OSA, compared to only 2.2% in the control group, yielding an odds ratio (OR) of 4.30 (95% CI: 3.75–4.94) (38). Even after adjusting for confounding variables, the association remained significant, with an adjusted OR of 1.60 (95% CI: 1.36–1.86).

Non-obstructive sleep disorders (nSD), including insomnia and hypersomnia, are also more frequent in HS patients, with 6.3% of HS patients affected compared to 1.8% of controls (OR: 3.63, 95% CI: 3.10–4.24) (38).

Although the mechanism remains unclear, the dysfunction of biological clock gene activates Th17-dominant skew (53). Therefore, sleep disturbance might become the trigger to enhance the development of Th17-mediated pathogenesis of HS.

Recognizing and addressing sleep disorders in HS patients is essential, as these conditions not only compromise quality of life but may also contribute to the progression of systemic complications. Early intervention offers a critical opportunity to mitigate these risks and improve overall patient outcomes.

## 4 The summary of interactions between lifestyle-related factors and HS

**Figure 1:**
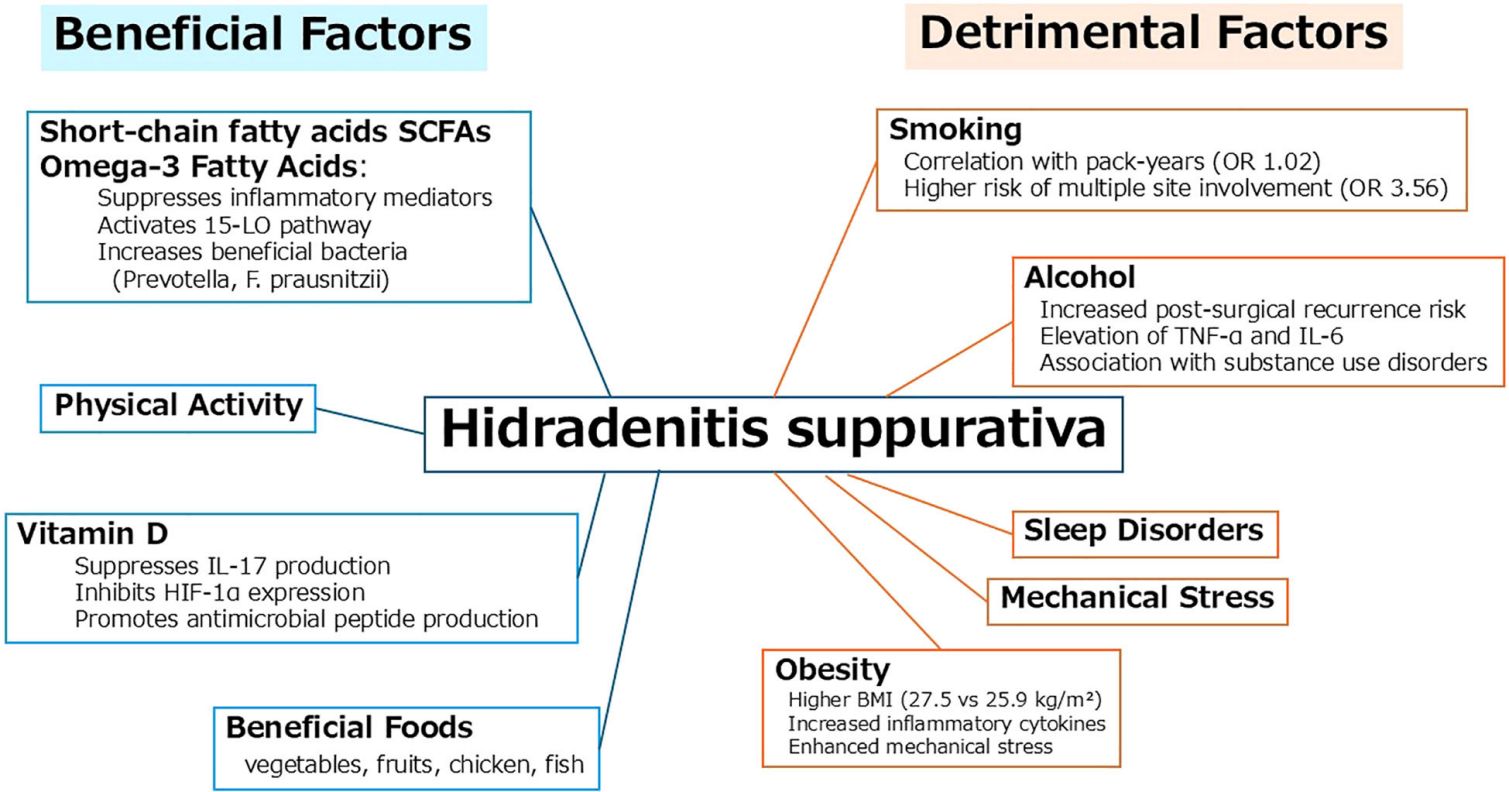
The interaction between HS and daily lifestyle.

HS is significantly influenced by various lifestyle-related factors. These factors can be broadly classified into beneficial and detrimental categories based on their impact on inflammation, immune modulation, and microbial balance (**Figure 1**).

Beneficial factors include short-chain fatty acids (SCFAs), omega-3 fatty acids, and vitamin D. SCFAs, derived from dietary fiber through gut microbial fermentation, contribute to the suppression of inflammatory mediators and support skin homeostasis. Omega-3 fatty acids modulate the immune response by activating the 15-lipoxygenase pathway and increasing beneficial gut bacteria such as *Prevotella* and *Faecalibacterium prausnitzii*. Vitamin D exerts anti-inflammatory effects by inhibiting IL-17 and HIF-1α expression and enhancing antimicrobial peptide production. Dietary patterns rich in vegetables, fruits, chicken, and fish are associated with these favorable effects.

In contrast, detrimental factors such as smoking, alcohol consumption, obesity, and sleep disorders have been linked to the onset and exacerbation of HS. Smoking shows a dose-dependent relationship with disease severity, including increased risk of multi-site involvement. Alcohol intake is associated with elevated TNF-α and IL-6 levels, and a higher risk of postoperative recurrence. Obesity contributes to disease progression through elevated inflammatory cytokines and mechanical stress in intertriginous areas. Sleep disorders may further exacerbate systemic inflammation and disrupt immune regulation.

Collectively, these interactions underscore the importance of lifestyle modification as an adjunctive approach to HS management. Promoting anti-inflammatory dietary habits while minimizing harmful exposures could potentially improve disease outcomes and enhance quality of life for affected individuals.

## 5 Conclusion

HS is a difficult dermatological condition that requires a multifaceted approach, encompassing both mechanistic understanding and lifestyle-based interventions. A holistic approach, including patient education on proper hygiene, clothing choices, stress reduction, and dietary considerations, may contribute to improved management and prevention strategies for this debilitating condition. Recognizing the impact of diet, stress, environmental exposures, and physical activity also allows for a more holistic approach to patient care. Lifestyle factors wield significant influence over the onset and progression of inflammatory skin disorders. Ongoing research is essential to elucidate the precise mechanisms and refine personalized approaches within the intricate landscape of these complex dermatological conditions.

This schematic representation of lifestyle-related beneficial and detrimental factors involved in the pathogenesis and progression of hidradenitis suppurativa (HS).

Beneficial factors such as short-chain fatty acids (SCFAs), omega-3 fatty acids, and vitamin D exert anti-inflammatory effects by inhibiting IL-17 and HIF-1α expression, promoting antimicrobial peptide production, and increasing beneficial gut bacteria. A healthy diet including vegetables, fruits, chicken, and fish is also supportive.

In contrast, detrimental factors such as smoking, alcohol consumption, obesity, and sleep disorders are associated with increased inflammatory cytokines, mechanical stress, and higher risk of disease progression and recurrence.
